# Implementation of a multi-level community-clinical linkage intervention to improve glycemic control among south Asian patients with uncontrolled diabetes: study protocol of the DREAM initiative

**DOI:** 10.1186/s12902-021-00885-5

**Published:** 2021-11-23

**Authors:** Sahnah Lim, Laura C. Wyatt, Shinu Mammen, Jennifer M. Zanowiak, Sadia Mohaimin, Andrea B. Troxel, Stacy Tessler Lindau, Heather T. Gold, Donna Shelley, Chau Trinh-Shevrin, Nadia S. Islam

**Affiliations:** 1 Department of Population Health, NYU Grossman School of Medicine, 180 Madison Avenue, New York, NY 10016 USA; 2 Departments of Obstetrics and Gynecology and Medicine-Geriatrics, The University of Chicago, 5841 Maryland Avenue MC 2050, Chicago, IL 60637 USA; 3 Department of Population Health, NYU Grossman School of Medicine, 550 First Ave, VZ30, 6th floor, New York, NY 10016 USA; 4 Department of Public Health Policy and Management Department, NYU Global School of Public Health, 665 Broadway, 11th Floor, New York, NY 10012 USA

**Keywords:** South Asian, Community health workers, Electronic health records, Diabetes management, Community-based participatory research, Health disparities, Structural determinants of health

## Abstract

**Background:**

A number of studies have identified patient-, provider-, and community-level barriers to effective diabetes management among South Asian Americans, who have a high prevalence of type 2 diabetes. However, no multi-level, integrated community health worker (CHW) models leveraging health information technology (HIT) have been developed to mitigate disease among this population. This paper describes the protocol for a multi-level, community-clinical linkage intervention to improve glycemic control among South Asians with uncontrolled diabetes.

**Methods:**

The study includes three components: 1) building the capacity of primary care practices (PCPs) to utilize electronic health record (EHR) registries to identify patients with uncontrolled diabetes; 2) delivery of a culturally- and linguistically-adapted CHW intervention to improve diabetes self-management; and 3) HIT-enabled linkage to culturally-relevant community resources. The CHW intervention component includes a randomized controlled trial consisting of group education sessions on diabetes management, physical activity, and diet/nutrition. South Asian individuals with type 2 diabetes are recruited from 20 PCPs throughout NYC and randomized at the individual level within each PCP site. A total of 886 individuals will be randomized into treatment or control groups; EHR data collection occurs at screening, 6-, 12-, and 18-month. We hypothesize that individuals receiving the multi-level diabetes management intervention will be 15% more likely than the control group to achieve ≥0.5% point reduction in hemoglobin A1c (HbA1c) at 6-months. Secondary outcomes include change in weight, body mass index, and LDL cholesterol; the increased use of community and social services; and increased health self-efficacy. Additionally, a cost-effectiveness analysis will focus on implementation and healthcare utilization costs to determine the incremental cost per person achieving an HbA1c change of ≥0.5%.

**Discussion:**

Final outcomes will provide evidence regarding the effectiveness of a multi-level, integrated EHR-CHW intervention, implemented in small PCP settings to promote diabetes control among an underserved South Asian population. The study leverages multisectoral partnerships, including the local health department, a healthcare payer, and EHR vendors. Study findings will have important implications for the translation of integrated evidence-based strategies to other minority communities and in under-resourced primary care settings.

**Trial registration:**

This study was registered with clinicaltrials.gov: NCT03333044 on November 6, 2017.

## Background

South Asian Americans are among the fastest growing ethnic groups in the U.S., and New York City (NYC) is one of the largest metropolitan areas for many South Asian groups, including Bangladeshis, Asian Indians, Nepalese, Pakistanis, and Sri Lankans [1–4]. Type 2 diabetes, a leading cause of death and morbidity in the U.S. and worldwide, disproportionately affects South Asians in the U.S. as well as in their home countries [5–7]. In NYC, data from the 2013–2015 NYC Community Health Survey estimated that 20% of South Asians self-reported a diabetes diagnosis compared to 11% of the overall sample [8]. Data from the 2004 NYC Health and Nutrition Examination Survey (NHANES) indicated that the prevalence of diabetes was 35.4% among foreign-born South Asians compared to 12.5% in the overall sample [9]. REACH U.S. Risk Factor Survey data from 2009 to 2012 found that 19.0% of Asian Indians self-reported a diabetes diagnosis, a rate higher than all other minority groups in the sample [10]. A cross-sectional study analyzing community-based cohorts in Chicago and San Francisco found that South Asians had a significantly higher prevalence of diabetes than all other ethnic groups: 23% vs. 6% among Whites [11].

Only a small number of studies have examined barriers to diabetes management among South Asian Americans. At the individual level, South Asian immigrants are shown to have poor knowledge of diabetes, are less likely to engage in physical activity compared to other racial and ethnic groups, and are more likely to consume high-fat foods after immigration to the U.S. [12–15]. Additionally, studies have demonstrated that South Asians are less likely to report receiving standard-of-care diabetes management compared to other groups. For example, a cross-sectional population-based study in NYC found that South Asian individuals were significantly less likely to receive eye examinations compared with Blacks [10]. It is likely that immigration status, lack of health insurance, mistrust of the healthcare system, and language and cultural barriers may help to explain poorer diabetes control among South Asian Americans [16].

In addition, studies have found that low self-efficacy and health-related social risks (e.g., low social support, food insecurity), are associated with higher levels of hemoglobin A1c (HbA1c), a key clinical marker for diabetes management [17–19]. In a study examining the relationship between perceived neighborhood social cohesion and diabetes/hypertension among South Asians, adjusted models found a significant association between high neighborhood social cohesion and reduced odds of having hypertension, while significance was not maintained for diabetes [20]. According to the U.S. Census, a number of social characteristics of the South Asian population may negatively affect diabetes management. For example, South Asians in NYC are more likely to live in poverty, have limited English proficiency, and lack access to culturally-appropriate community resources compared to the overall population [21, 22].

The U.S. Preventive Services Task Force has found sufficient evidence for three key strategies to improve diabetes management among patients: 1) multi-disciplinary team-based care [23]; 2) intensive lifestyle (diet or physical activity) interventions coupled with counseling [24]; and 3) identifying and tracking patients with diabetes through electronic health record (EHR)-based platforms according to risk and clinical practice guidelines. These strategies have had limited reach among South Asians. Despite the evidence for these interventions, a number of questions still remain, including who is the most effective member of the healthcare team to deliver lifestyle interventions (e.g. targeting physical activity or diet), what are the most effective strategies for coordinating within healthcare settings, and what is the effectiveness of these strategies among communities with limited English proficiency (LEP). Social determinants include the conditions “in which individuals are born, grow, live, work, and age” [25]. Substantial evidence supports the impact of social determinants on diabetes management and outcomes, which suggests that clinically-oriented interventions should be integrated with interventions that address an individual’s social context [17, 18]. It is important for physicians to consider contextual information about their patients in order to make correct decisions; strategies that address individualized clinical decisions are also needed [26, 27]. Our research was guided by the Chronic Care Model, which creates partnerships between health systems and communities, and by the NIMHD Minority Health and Health Disparities Research Framework, which addresses the complex nature of minority health disparities [28, 29]. This work shows that culturally-tailored and effective multi-level interventions to manage diabetes among South Asian Americans are sorely needed.

Community health workers (CHWs) act as a bridge between the community and the healthcare system. A substantial body of evidence demonstrates that CHWs are viewed as trusted sources of information and can be effective partners for dissemination of efficacious interventions to underserved communities as an intermediary of the health care system [30–32]. Additionally, a growing evidence base suggests that including CHWs in the primary care team is a cost-effective way to improve care among patients with chronic disease [33–36]. Our previous implementation work, informed by the RE-AIM (Reach, Efficacy/Effectiveness, Adoption, Implementation, Maintenance) framework [37, 38], has demonstrated that a culturally-adapted CHW-led intervention in community-based settings is acceptable and efficacious in improving HbA1c control, weight loss, self-efficacy and social support, and health behaviors among South Asian Americans with diabetes over a 6-month period [15, 39]. With the advent of the patient-centered medical home model, CHWs have played key roles in care management and referrals to community and social support services, such as English classes, insurance enrollment, and employment assistance, among other roles [40]. A recent meta-analysis of the impact of CHWs on diabetes management found that in nine pooled CHW interventions with at least 12 months of follow-up, the pooled effect size (standardized mean difference) which measures the incremental reduction in A1c by the intervention, above and beyond usual care, was 0.21 (0.11–0.32), measured in standard deviation units; the greatest reduction (> 0.5%) was seen among individuals with the highest initial HbA1c levels [30]. A 0.2% reduction in HbA1c is associated with 10% reduction in mortality; for clinicians, a difference of 5 mmol/mol (0.5%) between patient HbA1c samples is considered a significant change [41, 42]. In African American and Latinx communities, studies integrating CHWs into clinical teams have demonstrated even higher reductions in HbA1c [43]. The growing integration of CHWs into clinical settings to support team-based diabetes management for patients, along with reimbursement options for this workforce and the adoption of community resource referral technologies into clinical care [36, 40, 44], offer a unique opportunity to create multi-level sustainable, scalable models to meaningfully improve diabetes management, especially among marginalized populations.

EHR systems are increasingly being used to identify candidates for recommended follow-up [45] and targeted risk-reducing interventions [46–48], and can also be used to facilitate clinician referral of patients to a wide range of community-based programs and services [46–50]. The use of clinical decision support systems (CDSS), including alerts, can increase provider adherence to screening and monitoring guidelines for diabetes management and prevention, such as foot and retinal exams, as well as cholesterol and HbA1c testing [51–53]. EHR-based goal-setting modules, intention exercises, and tailored reminders to encourage behavior change of the patient during clinical encounters have enhanced provider capacity to counsel patients [54]. Studies have demonstrated that EHR systems and health information technology (HIT) strategies designed to identify, refer, and link patients to community resources, including social services, are feasible, acceptable, and effective [55, 56]. For example, an EHR-based e-prescribing model that linked patients to community resources according to patient need generated 253,479 personalized prescriptions for more than 113,000 patients in Chicago; most (83%) of the recipients found the referral very useful, and 19%, within about 2 weeks of receiving the prescription, reported using a new community resource [57]. In order to test the efficacy of this model, a scalable intervention was implemented in a prospective real-world clinical trial; confidence in finding resources increased for both groups over time, and this increase was significantly greater among the intervention group, with greater increases at each successive follow-up point [58]. To date, such models have not been widely implemented in small primary care practice (PCP) settings serving LEP immigrant communities. This gap suggests the need to further investigate the integration of referral systems into care for these populations.

Recent studies have demonstrated that EHR access and communication between the PCP and CHW can facilitate the acceptance and effectiveness of emerging care management models and lead to improved patient outcomes. Further, an emerging body of literature suggests that CHW and PCP efforts can be bolstered through integration of HIT tools that support referral to social services that address upstream determinants of health [40, 44, 59–61]. However, no studies have been conducted in small primary care settings focusing on South Asian American communities. Several studies have noted that integrating health systems, provider, patient, and community level intervention components may enhance the impact of diabetes management interventions [28, 62]; however, few have examined the impact of integrating multiple components and engagement of both physician and non-physician members of the healthcare team, including CHWs, in these efforts. Scalable and sustainable models that assess the integration and implementation of multi-level interventions in clinical practice settings are needed, particularly for immigrant and LEP populations such as the South Asian community.

This study protocol for the Diabetes Research, Education, and Action for Minorities (DREAM) Initiative, a multi-level diabetes management intervention designed to support South Asian patients with uncontrolled diabetes in NYC through clinic-community linkages, will support our aims to: 1) Test the effectiveness of a multi-level diabetes management intervention compared to usual care; and 2) Use a mixed-methods approach to systematically assess the implementation process and delineate factors influencing fidelity, adoption, and maintenance of the intervention within clinical and community settings. We hypothesize that compared to usual care, individuals receiving the multi-level diabetes management intervention will be 15% more likely to achieve at least a 0.5% reduction in HbA1c at 6 months. Secondary outcomes include a decrease in weight, body mass index (BMI), and LDL cholesterol; change in use of community and social services and self-efficacy related to healthcare, diabetes management, medication adherence, and health behaviors; and cost-effectiveness.

## Methods

### Study design

The intervention includes three components: 1) Building the capacity of PCPs to utilize EHR registry functions to identify patients with uncontrolled diabetes; 2) delivering a culturally- and linguistically-adapted CHW-intervention to improve diabetes self-management; and 3) HIT-enabled linkage to culturally-relevant community resources. A randomized controlled trial is being conducted for the CHW intervention component; South Asian individuals are currently being recruited from 20 PCPs throughout NYC. After screening and consent, individuals are randomized at the individual-level within each PCP site either to the CHW intervention (treatment) or to the usual care (control) group. The first year of the funded study was dedicated to recruiting PCP sites and adapting intervention components. During years 2–4, the EHR and CHW interventions are simultaneously being implemented, including HIT enabled linkage to community resources, and year 5 will be dedicated to dissemination efforts.

#### PCP recruitment and enrollment

We identified twenty independent PCPs in the boroughs of Queens and Brooklyn in NYC that are part of the Healthfirst network and serve a majority of South Asians (defined as > 70% of patients identifying as South Asians), by working in concert with community advisory board (CAB) members and building upon past successful collaborations [63]. Practices were required to use either eClinicalWorks or MDLand EHR platforms for 12 months before enrollment [64, 65]. New York University (NYU) Grossman School of Medicine study team staff contacted practice sites by telephone to assess practices’ eligibility for and interest in the study. If the practice representative expressed interest over the phone, the study team scheduled a site visit, during which eligible and interested practices signed a Memorandum of Understanding (MOU) for participation in the intervention.

#### Patient recruitment and randomization

**Fig. 1:**
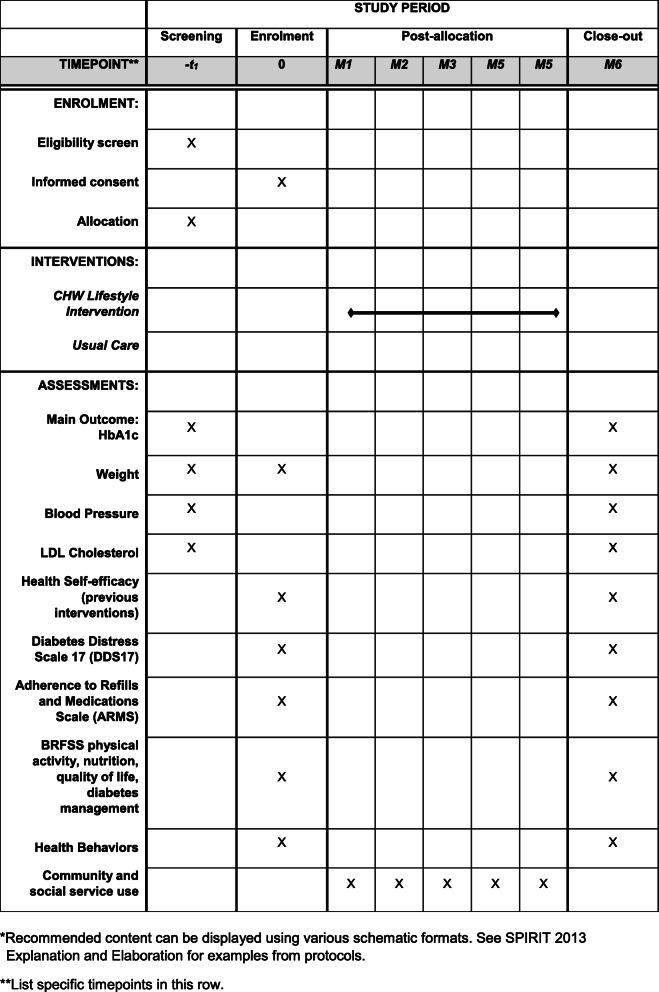
The schedule of enrolment, interventions, and assessments for the DREAM Intervention. *Recommended content can be displayed using various schematic formats. See SPIRIT 2013 Explanation and Elaboration for examples from protocols. **List specific timepoints in this row

Enrollment of the 20 PCP sites is roughly divided into three staggered waves over 3 years without overlap. Within each PCP site, individuals are randomized to the CHW intervention (treatment) or to the usual care (control) group. The study timeline is presented in Fig. 1.

The study team worked with EHR vendors to develop custom registry reports to systematically identify patients with uncontrolled diabetes using HbA1c values > 7% at the last clinic visit. Using this custom registry report, the study team is able to identify a list of eligible patients at each PCP site. Eligibility criteria include: age 21–74 years, not pregnant at the time of screening, HbA1c > 7.0% in the last 12 months, and has had a provider visit within the last 12 months. The data manager further cleans each EHR list for randomization by removing past study participants and individuals in ZIP codes too far from the PCP location (e.g. living outside of NYC or in a different borough that would require more than 1 h commute time). Potential family group members are identified using address and telephone numbers, as last names are often not the same for this community and often large family groups (e.g. siblings, parents, etc.) live together [66]; a randomization is performed for each family group, and one individual from each family group is randomly chosen and retained on the final list using the ‘sample’ command in base R.

Using the R software package ‘matching’ command, the data manager performs a matched pair randomization within each final PCP list, whereby a single individual is drawn from the list, and a matching control is found from the remaining individuals on the list based on age (within 0.5 standard deviation [SD]), sex (exact), and HbA1c (within 0.5 SD). If no matching control individual is identified, this individual is placed on an unmatched list. The matching process is repeated until everyone has either been matched or placed on the unmatched list. For Wave 1 a total of 6.4% of the eligible list was unmatched, and for Wave 2 a total of 8.1% of the eligible list was unmatched.

An invitation letter to participate in the CHW intervention, signed by their physician, is sent to all individuals randomized to the treatment; the CHW follows up a few days later with a telephone call. CHWs complete an in-person or phone-based screening with the individual to assess eligibility and session availability. Treatment participants sign a form as documentation of the informed consent process to participate; this is completed in-person by the CHW before the first session. Matched control group participants will never be contacted by the research team or participate in research activities (only their de-identified clinical data will be collected via the EHR) and receive usual care from their PCPs throughout study. In study Year 5, when all group sessions with treatment participants have ended, the control group participants will be offered diabetes-related group sessions as a point of service and not as a part of research.

### Study partners

The project is strengthened by a strong transdisciplinary Steering Committee consisting of academic, healthcare, and community partners that meets monthly to guide project design and implementation in all study phases. Our multi-sector partnership of community stakeholders includes Healthfirst, a not-for-profit HMO sponsored by 20 New York not-for-profit and public hospitals and systems serving more than 35,000 South Asians in NYC; thus the PCPs from the Healthfirst provider network who are enrolled into this study serve large South Asian communities [67]. Healthify is a leading software provider to health plans, hospitals, and provider networks working in low-income communities, allowing for quick and accurate referrals to social services for patients by care teams; Healthify provides reporting and analytics to understand the use and adoption of tools and referral completion. South Asian community-based organization (CBO) partners include the DREAM coalition, India Home, United Sikhs, Council of People Organization, Project New Yorker, and the South Asian Council on Social Services. Community partners will ensure the accuracy and comprehensiveness of community referral sources, support cultural and linguistic relevance of materials for the South Asian community, and provide venues for educational sessions. The CAB comprises South Asian CBOs who provide culturally-tailored expertise in the development and implementation of the CHW intervention, including reviewing and adapting the CHW curriculum and participant materials [39, 68].

### Ethics and data sharing protocols

The study protocol and procedures were approved by the Institutional Review Board at the NYU Grossman School of Medicine on November 27, 2017. All PCPs signed an MOU with NYU. The MOU’s responsibilities included: 1) EHR intervention components; 2) EHR training requirements; 3) de-identified EHR patient data extraction, confidentiality, and storage procedures; and 4) CHW intervention components. Written informed consent is obtained from all treatment group participants; control group participants are not consented because only their de-identified clinical data are collected via the EHR and they do not participate in any research-related activities. Consenting control group participants would otherwise create undue burden in terms of the CHW caseload. This CHW intervention component was registered via clinicalTrials.gov (NCT03333044) as of November 6, 2017.

### Intervention components

#### EHR capacity building

All 20 sites will receive the same EHR intervention, which was developed with input from project partners and participating PCP sites. The EHR intervention involves provision of support generating routine registry reports at each practice that identify patients with uncontrolled diabetes. The study will provide the practice with training on the following competencies: 1) understanding the functionality and potential impact of the registry; 2) generating registry reports for follow-up care; and, 3) identifying patients with uncontrolled diabetes.

In addition, participating PCP staff are trained on the importance of documenting vital signs and other pertinent health data, as well as how to utilize automated appointment reminder texts and letters that can be sent to patients electronically [69]. The study team provides an initial training to clinicians and/or staff members that primarily focuses on using the registry reports. The study team conducts follow-up technical assistance visits at each site every 2 months for up to 1 year after training to review initial training material and assist with troubleshooting the registry reports.

#### CHW intervention

##### CHW health coaching

The CHW intervention includes five monthly 60- to 90- min group health education sessions that provide the tools and strategies necessary to promote glycemic control and manage diabetes [39], held by paid, trained CHWs. The diabetes management curriculum is standardized and has been adapted from previous diabetes and hypertension management interventions [39, 63]. All sessions employ adult learning techniques and group-based learning and activities, and materials have been culturally adapted for diverse South Asian subgroups (including Asian Indian, Bangladeshi, Pakistani, and Indo-Caribbean communities) and linguistically-adapted into Bengali, Urdu, and Punjabi. The sessions also focus on risk factors that are culturally relevant for South Asian populations (see Table 1). Sessions are held in PCP offices and community spaces identified by partner agencies. Each session includes multiple timeslots in order to accommodate varying participant schedules. Between each session, CHWs follow up with participants by phone or in-person through a home or clinic visit for an initial action plan and follow-up progress notes; up to ten phone calls will be conducted throughout the intervention period. Two one-on-one in-person visits also take place between the participant and the CHW. These interactions are an opportunity for CHWs to discuss individualized challenges, strategies, and action plans for diabetes management. Participants engage in goal-setting activities related to diabetes management identified jointly by the patient and the CHW (e.g., medication adherence, diet, physical activity). Referral activities also happen during these interactions with CHWs and CHWs document them in the Healthify portal (see Third Component).

**Table 1:** Community Health Worker (CHW) Intervention Curriculum

Session Topic	Session Overview	Tailored Cultural Components
Session 1: Diabetes Overview	1. What is diabetes?	• Discussion of diabetes prevalence and increased risk of diabetes among South Asians
	2. Type I, Type II, Gestational Diabetes, Prediabetes	
	3. Risk factors	• Explanation of BMI and at-risk BMI in Asian communities
	4. Symptoms	• Dispelling common cultural misconceptions regarding diabetes
	5. Blood sugar (high/low)	
	6. Prevention of Diabetes, Diet, Exercise, Social Support and Goal Setting	
	7. Myths and Facts about diabetes	
Session 2: Nutrition	1. Eating a balanced diet	• Photos of typical South Asian foods
	2. Healthy eating tips	• Identifying and limiting sweets high in fat and sugar, and substituting sweets with fruits for dessert • Building a balanced plate following the Plate Method with traditional South Asian foods
	3. Overcoming barriers (e.g., eating out and in social situations)	• Managing expectations for eating outside the home • In language role play video on eating out in South Asian social setting
	4. Reading a Nutrition Label	• Using South Asian food labels
	5. Goal-setting for healthy eating	• Inclusion of the family cook within session /Working with family cook to improve nutrition in the entire household
Session 3: Physical Activity	1. Energy balance between foods and physical activity/ Calorie needs	
	2. Benefits of physical activity	• Discussion of the concept of “Saint-Soldier” in Sikhism, which promotes discipline in spiritual practice as well as in social responsibilities to family and community.
	3. Forms of exercise	
	4. Preventing injuries/safety	
	5. Incorporating physical activity routines and goal-setting	
	6. Overcoming barriers	• Home-based exercise/activities for women • List of free, local community exercise classes
Session 4: Stress Management	1. Effects of Stress on Physical and Emotional Health	
	2. Coping with different feelings	
	3. Stress Management Techniques	• Discussion around stigma associated with mental health problems such as depression • Herbal remedies for stress relief (e.g. fennel seed tea, ginger paste compress for the forehead)
	4. Family Support / Happy Family Relations	
Session 5: Diabetes Complications	1. Diabetes Overview	
	2. Heart Disease and Stroke	• Review of popular South Asian foods high in salt and fat and limiting these foods
	3. Managing Diabetes	• In language role play on receiving a prediabetes/diabetes diagnosis
	4. Staying motivated and goal-setting	

##### The COVID-19 pandemic and adaptations to CHW intervention

The COVID-19 shelter-in-place order in NYC took effect on March 16, 2020. The study had already completed the intervention for Wave 1 participants in January 2020. Wave 2 recruitment and enrollment began in August 2020. In response to the COVID-10 pandemic, the study was modified in these following ways: 1) in-person education sessions were provided remotely through telephone or video conferencing; 2) physical activity and other curriculum components were developed into videos and made available to participants via YouTube links; 3) curriculum material and weight scales were mailed to participants’ home address. These modifications may or may not be sustained for Wave 3 (tentatively planned for September 2021) depending on the progression of the pandemic and the public health and/or institutional policies in place at that time.

#### HIT-enabled referral to social services

Healthify is a cloud-based “search” platform that allows users to find services, filter through the resource and eligibility taxonomies, and refer patients to community services and programs for managing their social and behavioral health needs. Examples of service categories available on the Healthify portal include behavioral health, food, education, financial support, and housing.

##### Enhancement of the Healthify tool

For the first phase, the study team CHWs and the CAB worked with Healthify to enhance the referral portal to include culturally relevant resources for South Asians (e.g., job training programs provided in a South language), building upon community mapping activities already conducted.

##### User training

Healthify’s referral and tracking systems are designed to be used on mobile applications, and all CHWs utilize smartphones. Healthify engaged in a 10-h training for CHWs to use the platform, which included an overview of use, practice use sessions, and trouble-shooting. CAB members are onboarded as “network” partners, which enables both CAB members and CHWs to directly refer patients/clients within the established network. CAB members (“network” partners) received a 5-h training on how to use the platform, including management of referrals.

##### Closed loop referral system

Healthify conducts a workflow assessment with each of the network partners in order to establish a protocol and point person for receiving and documenting the outcome of the referrals. All study participants’ information is uploaded into the Healthify portal; when CHWs refer a patient to one of these network partners, the network partner receives a notification of the incoming referral. The network partner then reaches out to the referred patient and documents the outcome of the referral (e.g., patient eligibility was assessed) in the Healthify portal, thereby “closing the loop.”

### Sample size calculations

Sample size and power calculations were performed for the main outcome of interest – a reduction in HbA1c levels. Using a conservative estimate of a 10% difference in effect size (≥0.5% reduction in HbA1c), we require 443 individuals in each arm (treatment and usual care) to achieve 80% power using a standard 2-sided Type I error rate of 0.05. These calculations assume a 15% loss to follow-up and the enrollment and ongoing participation of 20 PCP sites.

#### Data collection

De-identified clinical measures for treatment and control group participants are extracted from EHR systems by the study team every 6 months for a period of 18 months (baseline, 6-, 12-, and 18-month time points). Study data are collected and managed using REDCap [70, 71] hosted at NYU Grossman School of Medicine. REDCap (Research Data Capture) is a secure, web-based software platform designed to support data capture for research studies. For the intervention group, non-clinical measures (e.g., self-efficacy) are collected via interviewer-administered baseline and endpoint surveys (6 months) and entered into REDCap. For the implementation evaluation, we will collect surveys with PCPs and in-depth interviews with CHWs, NYU research staff, CAB members, and PCPs. Study measures are detailed in Fig. 1.

The cost-effectiveness analysis will include implementation, intervention, and healthcare utilization costs. These costs include: 1) administering the program (e.g., phone calls, staff time associated with the recruitment process and the CHW intervention) based on CHW time surveys; 2) patient/family time estimated from visits and healthcare service use identified in Medicaid claims data (e.g., length of stay for hospitalization); and 3) cost of healthcare utilization by program participants based on Medicaid claims data and the Medicaid fee schedule for reimbursement amounts (e.g., hospitalizations, HbA1c tests). Healthcare utilization will allow for a 1 year follow-up.

#### Data analysis plan

All analyses will be conducted using R (R Foundation for Statistical Computing, Vienna, Austria). The primary outcome of interest is a reduction in HbA1c by ≥0.5% over the 6-month intervention period. The analysis of the effect of the intervention on the primary outcome measure will use a generalized linear mixed model (GLMM) with PCP-level random effects to account for clustering of patients within PCPs. Because control group participants are matched to intervention group participants on age and sex, all analyses will adjust for these covariates. CHW will be treated as a fixed effect in the models because there are not sufficient individual CHWs to measure statistical variation across CHWs. The analysis will be conducted on an intent-to-treat basis and will provide point estimates and 95% confidence intervals for the relative odds of achieving glycemic control; the confidence interval estimates will reflect both the pair matching and the clustering of patients within PCPs.

The secondary outcomes of interest include reductions in weight, BMI, and LDL cholesterol, referral to community services, and increased self-efficacy related to healthcare, diabetes management, medication adherence, and health behaviors. Analyses of secondary outcomes will use similar mixed effects regression models (adjusting for matching variables) to estimate the intervention effect. We will use the appropriate link function for the mixed effect model for continuous or binary outcomes.

For the implementation evaluation, we will conduct descriptive analyses using PCP survey data. Analysis of qualitative data from the in-depth interviews will follow techniques of narrative analysis and constant comparison analytic approach [72, 73]. The constant comparison approach is a method of explanation building in which the findings of an initial case are compared to a provisional category, property or proposition, and revised as necessary; other details or new cases are then compared against the revision and revised again as needed. We will develop an initial set of codes, informed by the open-ended questions, and information from the key informant interviews. For each core code, we will develop one or more secondary codes that represent either more specific or restricted aspects of the phenomenon, to contextualize it. The research team will code the interviews and discrepancies in coding will be discussed and resolved to achieve an acceptable level of inter-coder reliability. The coded transcripts will be analyzed with Atlas.ti [74].

For the cost-effectiveness analysis, we will estimate means, medians, and standard deviations for each cost category (implementation, intervention, and healthcare utilization) and assess categorical and total costs per patient at 6 and 12 months follow-up. Our outcome will be the incremental cost per person achieving a decrease of 0.5% in HbA1c.

## Discussion

While the efficacy of EHR and CHW interventions to promote diabetes management have been tested separately, evaluations testing these combined approaches are lacking. Our study proposes a unique integration of EHR and CHW approaches. Other studies have suggested that the integration of health systems and provider, patient, and community level components into diabetes management interventions may increase the impact on clinical outcomes [28, 62]. A limited number of studies have examined the impact of multiple intervention components. LEP populations, especially the South Asian community, need scalable and sustainable models that integrate multi-level interventions into clinical practice settings. Additionally, studies occurring in the context of small PCP settings with limited resources are important, and findings can potentially provide scalable and sustainable models for other minority communities that experience health disparities [75, 76]. Community-based practices serve a large percentage of immigrant and minority populations, especially in urban areas [77].

A few limitations should be mentioned. First, the network of PCPs is selected based on geographic clustering, so that the CHW workload can be managed through decreased travel between sites; this could result in participation bias. Second, individuals randomized to the control group do not receive the opportunity to accept or decline participation; however, when an individual randomized to the treatment group declines participation in the intervention, the control participant matched to them is also removed, thus removing some selection bias. Third, there is potential study contamination in these close-knit South Asian communities, as control group participants may interact with intervention participants or resources may be shared between intervention and control participants. We have attempted to reduce this bias by including only one likely family member, based on address and telephone number, during the randomization process, but some of this contamination may occur via non-family acquaintances, potentially biasing our results towards the null. Fourth, EHR data quality (e.g. weight) may differ by PCP site; in order to minimize this variation, we provide ongoing training and follow-up technical assistance to PCPs. Fifth, the provider may be positively influenced by components of the EHR intervention, including community resources shared with patients, which may affect his/her treatment and control participants alike. Lastly, because the CHW intervention was modified to be conducted remotely in response to the COVID-19 pandemic midway through the study timeline, the fidelity of the intervention is impacted. We will conduct sensitivity analyses to assess if there are any differences in the study outcomes based on intervention format. Related, the study’s primary outcome (i.e., reduction in HbA1c collected from the EHR) will likely have higher missingness for participants in Wave 2 and potentially Wave 3 because of interruptions in care during the pandemic. The study statisticians will explore analytic solutions such as multiple imputation for missing data and assess whether missingness is related to the study exposure and outcome.

This is the first study designed to test the effectiveness of a multi-level community-clinical linkage intervention among an underserved South Asian population in a real-world setting, utilizing paid CHWs. Evidence suggests that leveraging multisectoral partnerships should serve to enhance the study’s implementation and outcomes [78, 79]. Findings from this study have the potential to facilitate the translation of similar strategies across other minority communities, including Asian American, African American, and Hispanic American subgroups.

## Data Availability

The data and materials generated during this study are not publicly available, as the study is ongoing, but will be available from the corresponding author upon reasonable request.

## Abbreviations

BMI: Body mass index
CAB: Community Advisory Board
CHW: Community health worker
DREAM: Diabetes Research, Education, and Action for Minorities
EHR: Electronic Health Record
HbA1c: Hemoglobin A1c
HIT: Health Information Technology
MOU: Memorandum of Understanding
NYC: New York City
NYU: New York University
PCP: Primary care practice
